# A Simplified Process for Purification and Refolding of Recombinant Human Interferon-α2b

**DOI:** 10.52547/ibj.26.1.85

**Published:** 2021-12-04

**Authors:** Nima Hezarjaribi, Mohammad Reza Fazeli

**Affiliations:** 1 Department of Biology, Faculty of Basic Science, Islamic Azad University, Science and Research Branch, Tehran, Iran;; 2 Department of Drug and Food Control, Pharmaceutical Quality Assurance Research Center, Faculty of Pharmacy, Tehran University of Medical Sciences, Tehran, Iran

**Keywords:** Interferon alpha-2b, Inclusion bodies, Protein refolding

## Abstract

**Background:**

Interferon α-2b is a vital biotherapeutic produced through the recombinant DNA technology in *E. coli*. The recombinant IFN-α2b normally appears as intercellular IBs, which requires intensive refolding and purification steps.

**Method:**

Purification of IFN-α2b from solubilized IB was performed using two-phase extraction. To optimize refolding conditions, the effects of pH and different additives, including cysteine, cystine, urea, glycerol, Triton X-100, NaCl, and arginine, were investigated. Optimal refolding buffer (0.64 mM of urea, 5.57 mM of cysteine ​​, and 1.8 mM of cystine) was obtained using RSM. The refolding process was performed by an optimized refolding buffer in the dilution and fed-batch refolding method at different protein concentrations (25-1000 µg/mL).

**Result:**

At a final protein concentration of 500 µg/mL, the fed-batch refolding method yielded in a biological activity of 2.24 × 10^8 ^IU/mg, which was nearly twice that of dilution method.

**Conclusion:**

Fed-batch refolding method resulted in the biologically active IFN-α2b with high purity, which can be used for research and industrial purposes.

## INTRODUCTION

Interferon α-2b is a cytokine of the interferon I family involved in viral diseases. This protein is used for treating hepatitis B and C, as well as cancers such as multiple myeloma, chronic myeloid leukemia, renal cell carcinoma, epidermoid cervical cancer, non-Hodgkin's lymphoma, melanoma, medullary carcinoma thyroid, and head and neck cancers^[^^1^^]^.

*E. coli* is one of the IFN-α2b production hosts widely utilized in the biotechnology industry owing to the cost-effectiveness and high production levels. However, the overexpression of the target protein in cytoplasmic environment of* E. coli* often results in its accumulation as insoluble aggregates, called IBs, which inhibit the appropriate protein folding process^[^^2^^]^. Therefore, in a proper refolding method, different variables should be considered to achieve a high yield of refolded proteins. In general, after IB solubilization, the target protein is purified to remove the impurities^[^^3^^,^^4^^]^. Chromatographic methods are mainly applied to purify solubilized proteins such as ion exchange and size exclusion^[^^5^^]^. These methods are costly and time-consuming for protein purification. Therefore, by advancements in biotechnology, a two-phase extraction method using organic compounds, e.g. 2-butanol, is applied to separate and purify the protein. In this method, the total protein charge, solution buffer pH, and molecular protein weight are effective factors in protein migration to the organic or aqueous phase^[^^6^^]^. In two-phase extraction method, the target protein does purify as an unfold and inactive form, which is needed to be refolded to obtain its native structure. Refolding is the transformation of a protein from an unfolded to a folded state that occurs under optimal conditions of protein concentration, pH, appropriate additives at effective concentrations, and a proper temperature. The presence of denaturing agents used during the IB solubilization step unfolds the target protein; therefore, protein refolding occurs when the denaturant is removed. In this regard, a low concentration of denaturing agents would be present in the refolding buffer to keep the protein soluble^[^^7^^]^.

One of the standard methods for protein refolding is the dilution of solubilized protein in the refolding buffer. Low protein concentrations and reducing intermolecular protein interaction during the refolding process are necessary to decrease the accumulation of proteins and increase the refolding efficiency. In dilution method, an optimized refolding buffer is required to achieve high concentrations of refolded protein^[^^8^^]^. However, on a large scale, the dilution method is not cost-effective due to the high buffer volume consumption. In this regard, the fed-batch refolding method is applicable when the protein is highly concentrated. In fed-batch refolding, the protein is slowly added to the refolding buffer in the agitator reactor due to the inhibition aggregation in the refolding process. The fed-batch method advantages include the stability of active protein and the obtaining high final concentration of refolded protein^[^^9^^]^. 

In the present study, the recombinant IFN- α2b was isolated as IB. Following the IB solubilization, the purification of IFN-α2b was accomplished using a two-phase extraction method. Factors affecting the IFN-α2b refolding were screened and optimized by the design of experiment software. Finally, the achieved optimal condition was examined by both dilution and fed-batch methods.

## MATERIALS AND METHODS

High cell density culture of *E. coli *

*E. coli* BL21-DE3 recombinant carrying IFN-α2b expression cassette (Cell Bank of Zistdaru Danesh, Iran) was cultured and then inoculated into a fermenter (Eppendorf, USA). Fermentation was carried out as described before^[^^10^^]^. Cells were disrupted with a mechanical homogenizer at a pressure of 500 bar and the IBs were collected at 12000 ×g by centrifugation and were washed three times with PBS buffer.

Solubilization of IB 

IB was solubilized in a solubilizing buffer containing SDS 10%, EDTA (5 mM), DTT (50 mM), and Tris (50 mM) at pH 8. For each gram of IB, 10 ml of solubilization buffer was added.

Two-phase extraction

Two-phase extraction was achieved by using an organic-aqueous phase system with PBS^[^^6^^]^, which was then added to the solubilized protein at different pHs (3.5, 4.5, 5.5, 6.5, 7.5, and 8.5). Subsequently, 2-butanol was added to the solution and centrifuged (10,000 ×g for 12 min). The protein precipitated using 100 mM of sodium acetate at pH 6 (isoelectric point of IFN-α2b protein) and centrifugation at 10,000 ×g for 5 min. To obtain a soluble form of protein, the pellet was solubilized by using 6M guanidine with 2 mM of EDTA, 5 mM of DTT, and 50 mM of Tris at pH 8. 

Dilution refolding method in Plackett-Burman design

The Plackett-Burman experiment was used for screening additives by Minitab 2017 software^[^^4^^]^. The design was performed with eight factors, including pH^[^^11^^]^, cysteine, cystine, urea, glycerol, Triton X-100, NaCl, and arginine. Minitab 2017 software designed 12 experiments for the Plackett-Burman model, which was further analyzed using IFN-α2b antiviral bioassay data. After 24 h, the refolding reaction was stopped, and biological activity was measured using antiviral bioassay.

Refolding optimization 

The effective factors (urea, cysteine, and cystine) of the Plackett-Burman model were used in RSM designing. RSM design was carried out for optimal refolding buffer. Response surface was designed with full factorial, central composite, three factors, and six central repetition points. More additives extracted from Plackett-Burman, including 0.4 M of arginine, 5% glycerol, 50 mM of sodium chloride, 1 mM of EDTA, 50 mM of Tris buffer at an optimal pH 8, were added to experiment buffers. The refolding was performed by the dilution method at a concentration of 100 µg/mL protein for 24 hours, and the model was analyzed by the aid of Minitab software. After 24 h, the refolding reaction was stopped, and biological activity was measured using antiviral bioassay.

Fed-batch and dilution refolding methods

Different concentrations of IFN-α2b protein (25, 75, 150, 300, 500, and 1000 μg/mL) were tested in the fed-batch and dilution methods with an optimized refolding buffer. The fed-batch process was performed using a peristaltic pump with an inlet flow of 1 mL/5 min at 100 rpm agitator in a 350-mL Erlenmeyer reactor for 5 h. The protein refolding reaction was conducted at 4°C for 24 h. Furthermore, the dilution refolding method was carried out at the mentioned protein concentrations in an optimized refolding buffer. After 24 h, the refolding reaction was stopped, and the biological activity of the refolded protein was determined.

Antiviral bioassay

To perform the antiviral bioassay test, 100 μL of RPMI 1640 medium with 10% FBS was added to each well of a 96 sterile microtiter plate. The sample and NIBSC standard were separately diluted in culture medium at 40 IU, and 100 μL of diluted samples were added to the 96 sterile microtiter plate. Ten two-fold serial dilutions were performed onto the plate. Afterwards, the culture medium containing 5,000 A549 cells was added to each well. The tested plate was incubated at 37±1°C in a CO_2_ incubator for 20 h. Then Encephalomyocarditis virus was added to the plate and incubated for 40 h. The tested plate was stained with Amido black, and the absorption was measured by the use of an ELISA reader (BIOTEK, USA) at a wavelength of 610 nm. Thereafter, 4-parameter nonlinear regression analysis was performed by GEN5 2.09 software to calculate the biological activity of refolded IFN-α2b according to the biological activity of NIBSC standard. Refolding yield was calculated by dividing the biological activity of 1 mg of refolded IFN-α2b (IU/mg) to the biological activity of 1 mg of NIBSC.

**CD spectroscopy**

CD spectra were acquired with an AVIV 215 (AVIV, USA). A 0.1-cm path length quartz cell was used to record data between 180 and 240 nm with a 1-nm sampling interval. The refolded and IFN-α2b standard (Intron A) protein were desalted by an Amicon centrifugal filter (10 kDa cut-off) and diluted to 0.2 mg/mL.

## RESULTS

**Two-phase extraction at different pHs**

**Fig. 1:**
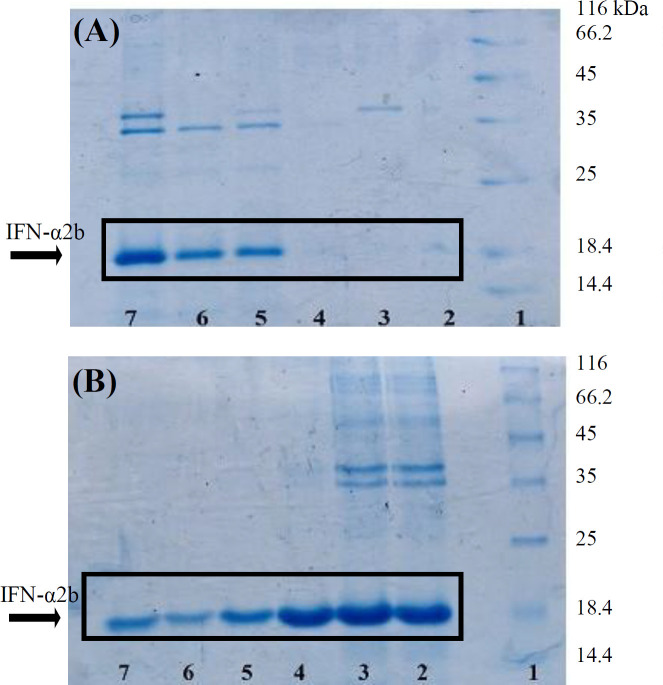
SDS-PAGE analysis of IFN-α2b purification by two-phase extraction method. The best IFN-α2b purity was obtained in the organic phase. The Figure shows the effect of different pHs of organic (A) and aqueous (B) phases on IFN-α2b purity, respectively. Lane 1, protein marker; lane 2, pH 3.5; lane 3, pH 4.5; lane 4, pH 5.5; lane 5, pH 6.5; lane 6, pH 7.5; lane 7, pH 8.5. IFN-α2b protein molecular weight is 19 kDa

The product of organic and aqueous phases separations at different pHs (3.5, 4.5, 5.5, 6.5, 7.5, and 8.5) were analyzed by SDS-PAGE^[^^12^^]^. As indicated in Figure 1, the highest amounts of IFN-α2b protein migrated to the organic were at pH 3.5 and 4.5, and for aqueous phases were at pH 6.5 and 7.5. However, many impurities were observed at these pHs. At pH 5.5, IFN-α2b protein was found in organic phase with very low amount of impurities.

**Screening and optimization of factors affecting refolding**

**Fig. 2:**
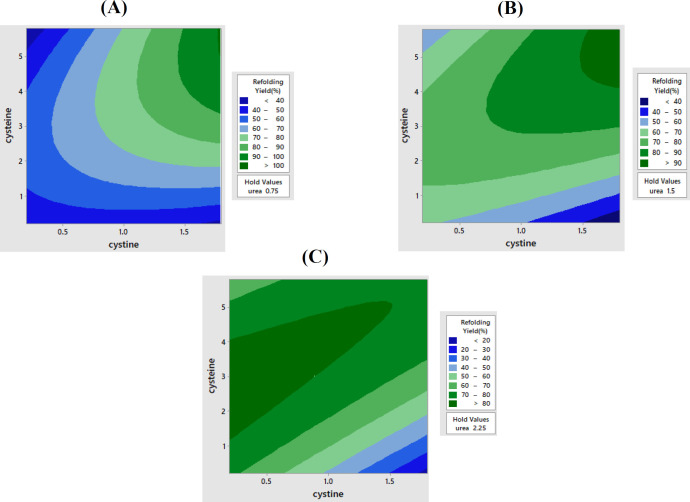
The analysis of different concentrations of cysteine/cystine and urea on IFN-α2b refolding by Minitab 2017 software. Urea concentration: (A) 0.75 M, (B) 1.5 M, and (C) 2.25 M; cysteine concentration: 1, 2, 3, 4, and 5 mM; cystine concentration: 0.5, 1, and 1.5 mM for A, B, and C

The results of 12 experiments of Plackett-Burman screening due to the *p* < 0.05, show the significant effect of each variable, including arginine, cystine, cysteine, urea, glycerol, and NaCl. The Plackett-Burman design and refolding yield are shown in Table 1. The IFN-α2b refolding efficiency was investigated by using the central composite RSM with six central repetition points in 20 experiments (Table 2). The optimal refolding buffer (0.64 mM of urea, 5.57 mM of cysteine ​​, and 1.8 mM of cystine) was obtained by RSM method. Furthermore, as depicted in Figure 2, the effect of cysteine and cystine in different concentrations of urea (0.75, 1.5, and 2.25 M) was studied. 

**Refolding using fed-batch and dilution method at high concentrations**

**Both Fed-batch and dilution methods were examined regarding the maximum protein concentration of refolded protein (25, 75, 150, 300, 500, and 1000 μg/mL) in the optimized refolding buffer. At 25, 75, and 150 μg/mL of IFN-α2b protein, the refolding yield was approximately the same in both methods. Furthermore, at 300, 500, and 1000 μg/mL of protein, the refolding yields in the dilution method were 65, 40, and 28, while those of the fed-batch method were 86, 80, and 61%.**

**Table. 1:** Plackett-Burman design of IFN-α2b refolding

**Experiment**	**pH**	**Sodium chloride** **(mM)**	**Triton** **X-100** **(%)**	**Glycerol** **(%)**	**Urea** **(M)**	**Cysteine** **(mM)**	**Cystine** **(mM)**	**Arginine** **(M)**	**Refolding yield of** **IFN-α2b**
1	9.5	300	0.125	5	0.5	4	0.4	1.0	75.44
2	9.5	300	0.250	5	0.5	1	2.0	0.4	64.50
3	9.5	50	0.250	5	2.0	4	0.4	1.0	69.60
4	9.5	50	0.250	15	2.0	1	0.4	0.4	59.20
5	7.5	50	0.125	5	0.5	1	0.4	0.4	90.40
6	7.5	50	0.125	5	2.0	1	2.0	1.0	48.80
7	9.5	50	0.125	15	0.5	4	2.0	0.4	81.60
8	7.5	300	0.250	15	0.5	1	0.4	1.0	61.20
9	7.5	50	0.250	15	0.5	4	2.0	1.0	62.40
10	7.5	300	0.125	15	2.0	4	0.4	0.4	64.80
11	7.5	300	0.250	5	2.0	4	2.0	0.4	52.80
12	9.5	300	0.125	15	2.0	1	2.0	1.0	25.60

**Secondary IFN-α2b structure analysis by CD**

**Fig. 3:**
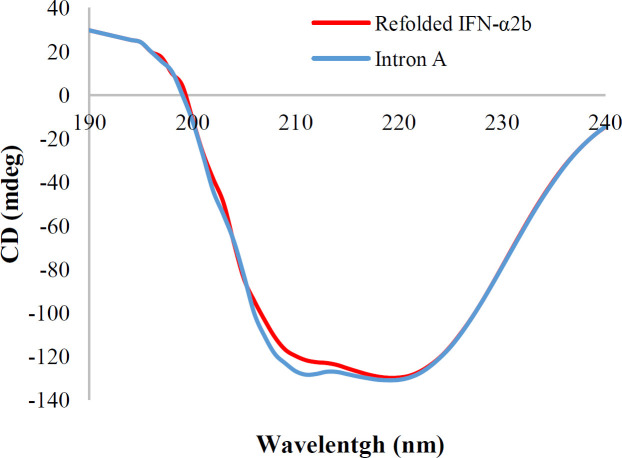
The far UV CD spectrum of IFN-α2b protein

The far UV CD spectrum of the IFN-α2b protein is demonstrated in Figure 3. Both sample and standard of IFN-α2b show a positive band at 193 nm and negative bands at 208 and 222 nm^[13]^, which are exactly what would be expected from a protein consisting of mainly α-helix structure.

## DISCUSSION

Two-phase extraction is an alternative method to chromatographic purification of some proteins. In this method, various factors, such as protein molecular weight, pH, and protein hydrophobicity affect protein migration to the organic or aqueous phase^[^^2^^]^. The obtained results indicated that the PBS buffer with pH 5.5 provided the best conditions for the extraction of IFN-α2b using the two-phase extraction method with high purity.

The Plackett-Burman method identified effective factors, including urea, cysteine, and cystine. RSM method was also used to optimize refolding buffer. According to the *p* value obtained from the RSM method, all factors were significant, and a suitable model was designed (data not shown). The smaller standard error indicates that the observations are close to the fitted line in the obtained model. R-square, with a value of 97.5% shows the model fits the data. The contour plot indicates the relationship between response and variables. Figure 2 displays that the optimal urea concentration for refolding of IFN-α2b is 0.75M. As a denaturing agent, urea promotes protein solubility by disrupting hydrophobic bonds and breaking hydrogen bond formation^[^^3^^]^. At low concentrations, urea stabilizes the protein structure, reduces protein interactions, and prevents aggregation^[^^7^^]^. The present study suggests that the ratio between cysteine and cystine is a critical factor for proper refolding. Basically, the molar ratios of 1:1 to 5:1 of reduced to oxidized thiol groups have been utilized for the proper folding of denatured proteins^[^^14^^,^^15^^]^. The results of this investigation demonstrated optimal refolding of IFN-α2b by using cysteine/cystine redox pairs is 3:1 of molar ratio.

**Table. 2:** RSM design of IFN-α2b refolding

**Experiment**	**Urea** **(M)**	**Cysteine** **(mM)**	**Cystine** **(mM)**	**Refolding yield of** **IFN- α2b (%)**
1	0.75	16.0	0.6	59.0
2	1.50	3.0	1.0	81.0
3	0.75	4.4	0.6	65.0
4	3.00	3.0	1.0	65.0
5	2.25	1.6	0.6	78.0
6	2.25	1.6	1.4	59.0
7	1.50	3.0	1.0	80.0
8	2.25	4.4	1.4	82.5
9	1.50	3.0	1.8	78.0
10	0.75	4.4	1.4	90.0
11	0.75	1.6	1.4	67.0
12	1.50	3.0	1.0	80.0
13	0.00	3.0	1.0	57.0
14	1.50	3.0	1.0	81.0
16	1.50	5.8	1.0	74.0
17	1.50	3.0	0.2	72.0
18	2.25	4.4	0.6	82.0
19	1.50	3.0	1.0	81.0
20	1.50	0.2	1.0	49.5

Initial high concentrations of protein in the refolding reaction can lead to a high concentration of folding intermediates, which leads to the formation of aggregated and misfolded proteins^[^^5^^]^. Therefore, to perform the refolding at high protein concentrations, the fed-batch method was used under controlled conditions. The fed-batch refolding method, at concentrations of 25, 75, 150, 300, and 500 μg/mL, resulted in a higher refolding yield than the dilution method. In IFN-α2b concentration of 1,000 μg/mL in fed-batch, refolding yield reduced due to aggregation. Refolding yield at low concentrations (150 μg/mL) was about 90% in the dilution method. At higher protein concentrations, owing to the rapid decrease of denaturant concentration^[^^16^^]^, the refolding yield decreased^[^^7^^]^. Using the fed-batch method, the amount of misfolded, aggregated and unfolded protein declined, even at high protein concentrations, and the refolding yield increased. Refolding of IFN-α2b with a concentration of 500 μg/mL using fed-batch method under optimized condition resulted in the refolding yield of 80% and biological activity of 2.24 × 10^8^ IU/mg, which shows this method is suitable for the production of IFN-α2b with high yield. Moreover, the results of the CD spectrum of standard and refolded IFN-α2b exhibited a high similarity in the secondary protein structure. 

Overall, the developed method for purification and refolding of IFN-α2b using two-phase extraction and fed-batch refolding is economical compared to chromatographic methods and dilution refolding, making this method promising for the industrial production of this valuable protein.

## CONFLICT OF INTEREST.

None declared.
